# Dose and Latency Effects of Leucine Supplementation in Modulating Glucose Homeostasis: Opposite Effects in Healthy and Glucocorticoid-Induced Insulin-Resistance States

**DOI:** 10.3390/nu4121851

**Published:** 2012-11-27

**Authors:** Nelo Eidy Zanchi, Lucas Guimarães-Ferreira, Mário Alves de Siqueira-Filho, Vitor Felitti, Humberto Nicastro, Carlos Bueno, Fábio Santos Lira, Marshall Alan Naimo, Patrícia Campos-Ferraz, Maria Tereza Nunes, Marília Seelaender, Carla Roberta de Oliveira Carvalho, François Blachier, Antonio Herbert Lancha

**Affiliations:** 1 Laboratory of Applied Nutrition and Metabolism, School of Physical Education and Sports, University of Sao Paulo, Sao Paulo, 05508-030, Brazil; Email: devfelitti@globo.com (V.F.); frnicastro@usp.br (H.N.); pacamos@usp.br (P.C.-F.); resjr@usp.br (A.H.L.); 2 Department of Physiology and Biophysics, Institute of Biomedical Sciences, University of Sao Paulo, Sao Paulo, 05508-900, Brazil; Email: lucas@cefd.ufes.br (L.G.-F.); deffmariofilho@gmail.com (M.A.S.-F.); kateresa@usp.br (M.T.N.); croc@icb.usp.br (C.R.O.C.); 3 Cancer Metabolism Research Group, Institute of Biomedical Sciences, University of São Paulo, Sao Paulo, 05508-900, Brazil; Email: yhfabioslira@gmail.com (F.S.L.); seelaenderrf@usp.br (M.S.); 4 Human Genome Research Center, Institute of Biosciences, University of São Paulo, São Paulo, 05508-900, Brazil; Email: carmao11@yahoo.com.br; 5 Department of Health Sciences and Human Performance, The University of Tampa, Tampa, FL 33606, USA; Email: mnaimo@ut.edu; 6 INRA, CRNH-IdF, UMR 914, Nutrition Physiology and Ingestive Behavior, Paris, 78350, France; Email: blachier@usp.br

**Keywords:** leucine supplementation, glucose homeostasis, skeletal muscle mass

## Abstract

Dexamethasone (DEXA) is a potent immunosupressant and anti-inflammatory agent whose main side effects are muscle atrophy and insulin resistance in skeletal muscles. In this context, leucine supplementation may represent a way to limit the DEXA side effects. In this study, we have investigated the effects of a low and a high dose of leucine supplementation (via a bolus) on glucose homeostasis, muscle mass and muscle strength in energy-restricted and DEXA-treated rats. Since the leucine response may also be linked to the administration of this amino acid, we performed a second set of experiments with leucine given in bolus (via gavage) *versus* leucine given via drinking water. Leucine supplementation was found to produce positive effects (e.g., reduced insulin levels) only when administrated in low dosage, both via the bolus or via drinking water. However, under DEXA treatment, leucine administration was found to significantly influence this response, since leucine supplementation via drinking water clearly induced a diabetic state, whereas the same effect was not observed when supplied via the gavage.

## 1. Introduction

Branched-chain amino acids (BCAA—leucine, isoleucine and valine) supplementation, especially leucine, has been described as a potential therapeutic tool capable to attenuate skeletal muscle atrophy induced by several catabolic conditions, such as cancer, sepsis, muscular diseases [1] and glucocorticoid treatment [2]. Since leucine is considered as the second more potent insulin secretagogue amongst all the amino acids (AA) [3], it has been studied for its capacity to modulate whole body glucose homeostasis [4]. 

Indeed, leucine supplementation might exert positive systemic effects in conditions characterized by increases in glucose homeostasis disturbance, such as high fat diet (HFD) induced insulin resistance, but the effects are controversial. For example, Zhang and coworkers [5] found increases in the glucose metabolism of leucine supplemented mice, whereas Lynch and coworkers [6] did not observe improvements nor decreases in the glucose homeostasis. However, this may have been due to the lack of standardization of doses and forms of administration. On the other hand, in healthy rats and humans, oral leucine feeding has shown to rapidly inhibit skeletal muscle protein degradation [7,8] and also promote robust increases in skeletal muscle protein synthesis [9], demonstrating overall a potential capacity to handle disturbances in glucose metabolism and spare skeletal muscle mass, especially under atrophic conditions [8]. However, its chronic effects remain elusive, specifically during insulin resistant states from different physiopathological backgrounds, such as glucocorticoid treatment (and even HFD treatment). 

Dexamethasone (DEXA) is a synthetic glucocorticoid form of the endogenous hormone cortisone, which exhibits potent immunosupressant and anti-inflammatory properties [10]. The successful therapeutic benefits of this drug in a wide range of inflammatory diseases is, however, limited as it presents several side effects [11], such as insulin resistance and skeletal muscle atrophy [12,13]. Therefore, in order to benefit from the desired effects of long term DEXA treatment, the deleterious responses must be reduced. In this context, leucine supplementation may represent an interesting intervention with a clinical perspective, due to the reasons justified from above. 

Since there is still a lack of evidence regarding the effects of leucine supplementation on DEXA-induced insulin resistance and skeletal muscle atrophy, we decided to investigate: (1) whether a supplementation, with a low dose of leucine (not capable of increasing insulin levels or muscle protein synthesis) or a high dose of leucine (capable of maximally increasing both insulin concentration and muscle protein synthesis) given through gavage or drinking water is able to improve glucose metabolism, as well as spare the muscle mass and, consequently, voluntary muscular strength in healthy (control pair-fed and energy restricted) rats; (2) investigate to see if leucine supplementation via gavage or via drinking water exerts some positive effect on glucose metabolism and muscle sparing/strength effects under DEXA treatment; in other words, besides the dosage effect, would frequent nutritional stimuli (leucine provided through drinking water) be different from that provided by a pulsatile pattern (leucine provided through gavage) with both groups consuming the same daily dosage? 

## 2. Materials and Methods

### 2.1. Animals

The experiments were conducted in accordance with the National Research Council’s Guidelines for the Care and Use of Laboratory Animals. All methods used were approved by the Ethical Committee for Animal Research of the Physical Education and Sport of the University of Sao Paulo (protocol 2008/45). Adult male Wistar rats (mean body weight 440 g) were housed in individual cages under controlled environmental conditions (temperature, 22 °C; 12-h dark period) with a standard diet (Nuvilab, Brazil) and water provided *ad libitum*.

### 2.2. Study 1

#### Groups

Animals were randomly divided into the following groups: control non-supplemented (CON-NS; *n* = 10), control + leucine low-dose (CON-LL; *n* = 10), control + leucine high-dose (CON-LH; *n* = 10), DEXA (DEX; *n* = 10), DEX + leucine low-dose (DEX-LL; *n* = 10) and DEXA + leucine high-dose (DEX-LH). During the duration of the experiment, which lasted seven days, DEXA (a synthetic glucocorticoid analogue that does not bind to plasma binding proteins) was given daily (at 9:00 a.m.) through intraperitoneal injection (5 mg/kg/day); control groups received an equivalent volume of saline (0.9% NaCl). As DEXA was reported to decrease food intake, all groups were fed the same amount of food (in terms of caloric intake) equal to the DEX group. Thus, differences among groups did not originate from different food intakes. We measured the caloric content of our standard chow (16.32 kJ/g) as well as leucine (25 kJ/g) in a calorimetric bomb (FTT Oxygen Bomb Calorimeter) in order to avoid differences in the caloric ingestion between experimental groups and observed that the total caloric consumption was not statistically different among groups. A suspension of 54.0 g of L-leucine/L in water was prepared according to Crozier and coworkers [9]. Rats of leucine-supplemented groups received 0.068 g/kg/day (low-dose) or 1.35 g/kg/day (high-dose) twice a day (8:00 a.m. and 2:00 p.m.) through gavage during seven days [9]. Importantly, the high dosage were capable of maximally increasing muscle protein synthesis and insulin plasmatic levels (in a well defined pulsatile form), whereas the low dosage was not capable of increasing either muscle protein synthesis or insulin plasmatic levels [9,14]. Non-supplemented groups received 0.155 mol/L of NaCl at a volume of 2.5 mL/100 g of body weight twice a day. This volume of saline is equivalent to the volume of leucine suspension administered to leucine-supplemented groups and was chosen in order to take into account any possible volume-induced effects of oral gavage, *i.e.*, gastric expansion-induced signaling. We chose to administer two daily doses of leucine in order to maintain plasma increased concentration throughout the day and counteract DEXA-induced effects. Animals were euthanized after 13 h fasting by decapitation. Soleus and extensor digitorius longus (EDL) muscles of each limb were isolated, weighed and frozen at −80 °C for analysis. To assess the dry over total weight ratio, a small portion of each muscle was weighed and then dried for 48 h. 

### 2.3. Study 2

Since we observed that control groups responded equally to leucine supplementation via bolus or drinking water (data not shown), we additionally investigated whether or not leucine supplementation would be different via a bolus or drinking water in the presence of DEXA treatment. 

#### 2.3.1. Groups

Animals were randomly divided into the following groups: DEXA + leucine low-dose (DEX-LL; *n* = 8); DEXA + leucine low-dose drinking water (DEX-LL-H_2_O; *n* = 8); DEXA + leucine high-dose (DEX-LH; *n* = 8); and DEXA + leucine high-dose drinking water (DEX-LH-H_2_O; *n* = 8). The groups received the same dosage of DEXA administered in study 1. All groups were the same amount of food (in terms of caloric intake) equal to the DEXA group, and no statistically differences among groups were observed. DEX-LL and DEX-LH groups were supplemented via gavage (0.068 and 1.35 g/kg/day, respectively) twice a day and fed the same diet with regular tap water as drinking water, as previously described in study 1. DEX-LL-H_2_O and DEX-LH-H_2_O groups were supplemented with leucine via drinking water, and the leucine dose was adjusted every day on the basis of drinking water intake the day before. The water or liquid leucine supplement was provided by means of graduated cylinders topped with a 1-hole rubber stopper holding a metal drinking nipple. Leucine in water solution was used as crystals grounded to a fine powder with a ceramic mortar and pestle to optimize solubility [15]. In this study, we added leucine in drinking water to compare leucine provided through gavage (which results in a well defined pulsatile pattern [9,14]) *versus* leucine provided through drinking water (rendering less fluctuations in the leucine levels) with both groups consuming the same daily dosage. Animals were euthanized by decapitation and soleus, and EDL muscles of each limb were isolated, weighed and frozen at −80 °C for analysis. 

#### 2.3.2. Basal Fasting Glucose, Insulin and Tryacilglycerol (TAG) Levels

Basal and fed glycemia were measured through blood collected from the caudal vein after an overnight fast (13 h) using a digital glucometer (ACCU-CHEK Performa, Roche) before euthanasia. Immediately after euthanasia (13 h fasting), blood was collected and serum samples were prepared on ice. Serum was frozen and stored at −80 °C for analysis. Basal TAG was measured using a commercial kit (Biolab, Brazil). Serum insulin concentration was quantified using the commercial kits RIA (DPC^®^, Brazil). The homeostase model for assessment of insulin resistance index (HOMA-IR) was calculated as follows: 

HOMA-IR index (mmol·mU/L^2^) = fasting insulin (mU/L) × serum glucose (mmol/L)/22.5 [16].

#### 2.3.3. Motor Performance Tests

In order to evaluate skeletal muscle function, two evaluations were carried out. Such evaluations are widely adopted as measures of muscle function in dystrophic mice. The first one, the Grip Strength System Test (model: DFE-002, San Diego Instruments, San Diego, CA, USA) is a condition where animals are let to grab onto the system with the forepaws as the experimenter gently pulls on their tails. This allows the experimenter to determine the maximal strength before the animal releases the bar [17]. Importantly, all measurements of maximal strength were performed by the same investigator, who was highly experienced with performing this test.

The second motor performance test is the ambulation test. This test allows the determination of the mean length of a step measured in hindfoot ink prints and is normalized by the animal’s length. Briefly, rats were allowed to freely run in a corridor (length, 100 cm; width, 10.5 cm; height of lateral walls, 20 cm) three different times. Before the test, the animals were permitted to explore the apparatus [18,19]. Mean values were individually calculated for each test through the mean of three consecutive tests performed during one minute.

#### 2.3.4. RNA Isolation and Realt-Time PCR

Total RNA was extracted from homogenized soleus and EDL muscles with the Trizol reagent (Invitrogen) according to the manufacturer instructions. One microgram of total RNA was retranscribed with MMLV enzyme (Invitrogen), and an aliquot was used to measure real-time PCR. All reactions were conducted in a volume of 25 µL containing 4 mM MgCl_2_ (Invitrogen), 0.25 mM dNTPs (Invitrogen), 1.2 U of Taq polymerase (Invitrogen), 1/30000 Sybr Green (Invitrogen) and specific oligonucleotides for each gene with the Rotor Gene 3000 sequence detector (Quiagen Inc.; Hilden, Germany). Primers utilized for real-time PCR analysis was: GLUT-4 sense: 5′-GGGCTGTGAGTGAGTGCTTTC-3′; antisense: 5′-CAGCGAGGCAAGGCTAGA-3′; GAPDH sense: 5′-GATGGGTGTGAACCACGAGAAA-3′; antisense: 5′-ACGGATACATTGGGGGTAGGA-3′. Reactions were run for 40 cycles under the following conditions: 40 s at 95 °C, 40 s at 65 °C and 40 s at 72 °C. The amplification of unique products in each reaction was verified by melting curve and ethidium bromide (Sigma Aldrich) stained agarose gel electrophoresis. Each sample was run in triplicate. The expression level of each gene was normalized to housekeeping gene (GAPDH) expression level using the standard curve method. Mean and standard errors were calculated and are expressed as fold changes relative to the control group.

#### 2.3.5. Statistical Analysis

The dependent variables were tested by either one-way or two-way ANOVA, as appropriate. A *post-hoc* test with a Tukey adjustment was performed for multiple comparison purposes. The significance level was set at *p* < 0.05. The results are expressed as means ± S.E.M. 

## 3. Results

### 3.1. Study 1

The effects of DEXA treatment and leucine supplementation on body weight and muscle morphological parameters: as shown in Table 1, the baseline body weight was similar among groups. All groups were characterized by a significant body weight reduction at the end of the experimental protocol (*p* < 0.05). DEXA-treated groups showed significantly reduced body weight when compared with the control groups (*p* < 0.05). Thus, leucine supplementation at both low and high doses did not counteract body weight loss in both food restricted (control groups) and DEXA-treated animals. Soleus muscle mass did not differ among groups. Leucine supplementation at high doses attenuated food restriction-induced EDL muscle loss (CON-LH group) when compared with the CON-NS group (*p* < 0.05). All DEXA-treated animals presented reduced EDL muscle mass when compared with the CON-NS group (*p* < 0.05), and leucine supplementation at both low and high doses of amino acid did not attenuate it (total *n* = 60).

**Table 1:** Body and muscle morphological parameters of the experimental groups.

Variable	Group					
	CON-NS	CON-LL	CON-LH	DEX-NS	DEX-LL	DEX-LH
Initial BW (g)	442.7 ± 5.90	442.2 ± 4.91	441.0 ± 4.35	443.8 ± 6.19	443.3 ± 4.19	444.8 ± 6.16
Final BW (g)	381.9 ± 22.1 ^b^	386.3 ± 20.32 ^b^	375.0 ± 10.21 ^b^	343.4 ± 11.53 ^a,b^	345.79 ± 15.89 ^a,b^	339.9 ± 11.73 ^a,b^
Delta BW (g)	−60.8 ± 4.64	−55.9 ± 5.36	−65.17 ± 4.98	−100.8 ± 2.40 ^a^	−103.6 ± 2.08 ^a^	−104.5 ± 3.35 ^a^
Soleus (mg)	214.8 ± 5.99	223.3 ± 5.45	221.0 ± 6.13	214.4 ± 4.29	210.8 ± 2.25	212.7 ± 5.45
EDL (mg)	189.4 ± 2.31	197.3 ± 2.08	200.1 ± 1.49 ^a^	179.6 ± 3.37 ^a^	174.8 ± 2.80 ^a^	174.6 ± 4.12 ^a^

Values are expressed as mean ± SE. Control non-supplemented group (CON-NS; *n* = 10); Control leucine supplemented group with low dose via gavage (CON-LL; *n* = 10); Control leucine supplemented group with high dose via gavage (CON-LH; *n* = 10); DEXA non supplemented group (DEX-NS; *n* = 10); DEXA treated group plus low dose of leucine supplementation via gavage (DEX-LL; *n* = 10); DEXA treated group plus high dose of leucine supplementation via gavage (DEX-LH; *n* = 10). BW—body weight; EDL—extensor digitorum longus. ^a^ *p* < 0.05 *vs.* CON-NS; ^b^ *p* < 0.05 *vs.* Initial BW.

The effects of DEXA treatment and leucine supplementation on water intake: In the control groups, water intake was significantly increased in CON-LL group when compared with the CON-NS group at day 3 (38.33 ± 0.57 in CON-LL group *vs.* 29.00 ± 2.93 in CON-NS group; *p* < 0.05). At day 6 of treatment, DEX-NS group showed increased water intake when compared with day 1 (36.77 ± 2.34 in DEX-NS group at day 6 *vs.* 28.09 ± 2.05 at day 1; *p* < 0.05) and with the CON-NS group at day 6 (27.53 ± 2.68 in CON-NS group at day 6; *p* < 0.05). DEX-LL group presented reduced water intake at day 3 when compared with day 1 (23.14 ± 1.04 in DEX-LL group at day 3 *vs.* 31.2 ± 2.05 at day 1; *p* < 0.05) and increased when compared with the CON-NS group at day 6 (*p* < 0.05) (total *n* = 60).

**Figure 1:**
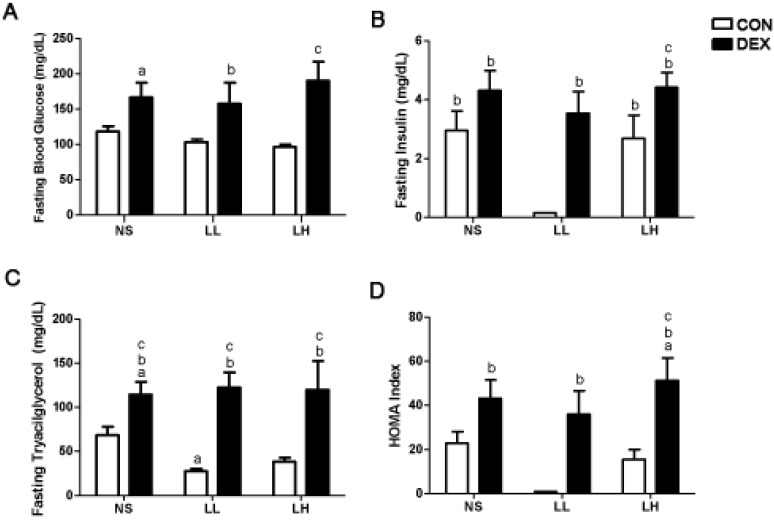
Fasting (**A**) blood glucose, (**B**) insulin, and (**C**) triacylglycerol levels and (**D**) HOMA index. Values are expressed as mean ± S.E.M. ^a^ Different from CON-NS (*p* < 0.05); ^b^ Different from CON-LL (*p* < 0.05); and ^c^ Different from CON-LH (*p* < 0.05).

The effects of DEXA treatment and leucine supplementation on serum glucose, insulin and triacylglycerol (TAG): fasting glycemia and tryacilglycerol were significantly higher in the DEX-NS group when compared with the CON-NS group (Figure 1A; 166.6 ± 20.8 mg/dL in DEX-NS *vs.* 118.5 ± 7.5 mg/dL in CON-NS group; *p* < 0.05; Figure 1C; 114.4 ± 14.4 mg/dL in DEX-NS *vs.* 68.3 ± 9.7 mg/dL in CON-NS group; *p* < 0.05), suggesting a DEXA-induced increase in blood glucose and TAG. Leucine supplementation at a low dose decreased fasting insulin and triacylglycerol when compared to the CON-NS and CON-LH groups (0.15 ± 0.01 mg/dL in CON-LL *vs.* 2.95 ± 0.66 mg/dL in CON-NS and 2.68 ± 0.79 mg/dL in CON-LH groups; *p* < 0.05; Figure 1B; 27.56 ± 2.89 mg/dL in CON-LL *vs.* 68.31 ± 9.74 mg/dL in CON-NS group; *p* < 0.05; Figure 1C). DEX-LL and DEX-LH groups presented increased fasting glycemia (157.6 ± 29.8 mg/dL in DEX-LL *vs.* 103.3 ± 4.0 mg/dL in CON-LL group; *p* < 0.05; 190.1 ± 27.2 mg/dL in DEX-LH *vs.* 96.7 ± 3.2 mg/dL in CON-LH group; *p* < 0.05; Figure 1A), insulin (3.54 ± 0.74 mg/dL in DEX-LL *vs.* 0.15 ± 0.01 mg/dL in CON-LL group; *p* < 0.05; 4.42 ± 0.51 mg/dL in DEX-LH *vs.* 2.68 ± 0.79 mg/dL in CON-LH group; *p* < 0.05; Figure 1B) and triacylglycerol (122.4 ± 16.9 mg/dL in DEX-LL *vs.* 27.6 ± 2.9 mg/dL in CON-LL group; *p* < 0.05; 119.7 ± 32.7 mg/dL in DEX-LH *vs.* 38.5 ± 4.7 mg/dL in CON-LH group; *p* < 0.05; Figure 1C) when compared to its respective control groups. Thus, it is possible to emphasize that these effects were meditated by leucine since both groups had the same food intake (*n* = 10 per group).

In the fed state, control groups did not show any significant alteration in serum glucose during the experimental protocol. However, DEXA-treated groups showed markedly increased serum glucose levels at days 3 and 6 when compared with day 1 (*p* < 0.05) and with the CON-NS group at days 3 and 6 (*p* < 0.05). No significant differences were observed among DEXA-treated groups (Table 2) (*n* = 10).

**Table 2:** Fed serum glucose (mg/dL) of the experimental groups on days 1, 3 and 6 of the study.

Day	Group					
	CON-NS	CON-LL	CON-LH	DEX-NS	DEX-LL	DEX-LH
1	110.5 ± 5.73	107.3 ± 0.76	115.5 ± 1.47	120.1 ± 5.06	104.8 ± 4.07	114.7 ± 4.78
3	109.8 ± 5.26	103.5 ± 1.16	129.6 ± 1.28	215.6 ± 29.08 ^a,b^	196.5 ± 17.27 ^a,b^	178.1 ± 15.18 ^a,b^
6	109.8 ± 5.26	110.0 ± 1.64	130.7 ± 2.25	267.3 ± 31.51 ^a,b^	255.2 ± 23.44 ^a,b^	215.6 ± 22.33 ^a,b^

Values are expressed as mean ± SE. Control non-supplemented group (CON-NS; *n* = 10); Control leucine supplemented group with low dose via gavage (CON-LL; *n* = 10); Control leucine supplemented group with high dose via gavage (CON-LH; *n* = 10); DEXA non supplemented group (DEX-NS; *n* = 10); DEXA treated group plus low dose of leucine supplementation via gavage (DEX-LL; *n* = 10); DEXA treated group plus high dose of leucine supplementation via gavage (DEX-LH; *n* = 10). ^a^ *p* < 0.05 *vs.* CON-NS in the same day of treatment; ^b^ *p* < 0.05 *vs.* day 1 in the same group.

**Figure 2:**
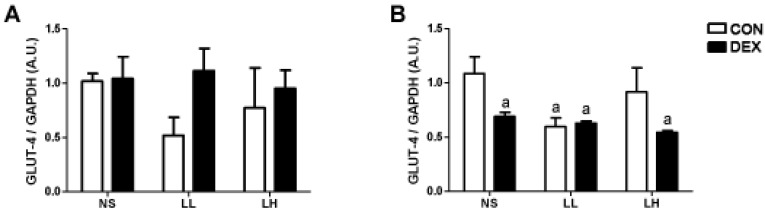
GLUT-4 gene expression in (**A**) soleus and (**B**) EDL muscles Values are expressed as mean ± S.E.M. ^a^ Different from CON-NS (*p* < 0.05).

The effects of DEXA treatment and leucine supplementation on GLUT-4 gene expression in skeletal muscle: in soleus muscle, GLUT-4 gene expression was not significantly affected by DEXA or leucine treatment. In EDL muscle, however, GLUT-4 mRNA content was significantly lower in DEX-NS, DEX-LL and DEX-LH groups (0.69 ± 0.38, 0.63 ± 0.18 and 0.54 ± 0.17, respectively) when compared to CON-NS group (1.00 ± 0.15; *p* < 0.05; Figure 2A). In control supplemented groups, only CON-LL was lower than CON-NS (0.60 ± 0.16 *vs.* 1.00 ± 0.15; *p* < 0.05; Figure 2B) (*n* = 6–8 animals per group).

**Figure 3:**
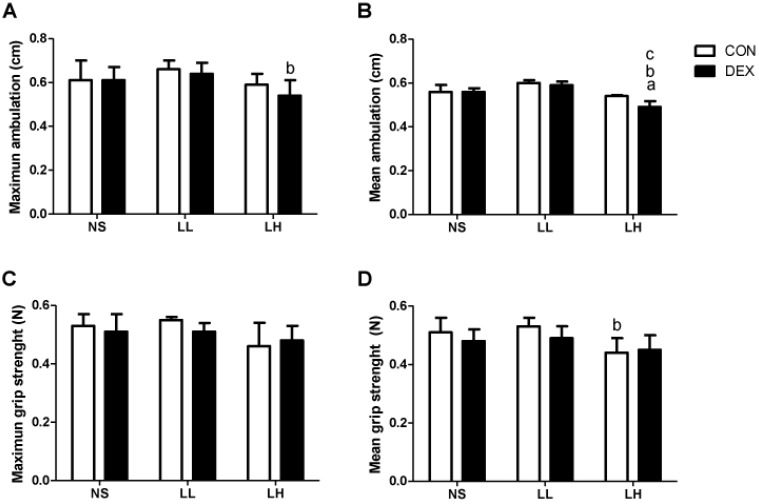
Muscle functional parameters. (**A**) Maximum ambulation (cm); (**B**) Mean ambulation (cm); (**C**) Maximum grip strength (N); (**D**) Mean grip strength (N). Values are expressed as mean ± S.E.M. ^a^ Different from CON-NS (*p* < 0.05); ^b^ Different from CON-LL (*p* < 0.05); and ^c^ Different from CON-LH (*p* < 0.05).

The effects of DEXA treatment and leucine supplementation on muscle functional parameters: We observed that DEX-LH animals presented a modest but significant deficit in mean ambulation when compared to the CON-LH group (0.54 ± 0.03 cm in DEX-LH *vs.* 0.59 ± 0.02 cm in CON-LH group; *p* < 0.05; Figure 3B). The CON-LH group also presented less mean grip strength when compared with the CON-LL group (0.46 ± 0.04 N in CON-LH *vs.* 0.66 ± 0.02 N in CON-LL; *p* < 0.05; Figure 3D) (*n* = 8–10 per group).

### 3.2. Study 2

The effects of DEXA treatment and leucine supplementation on body weight and muscle morphological parameters: baseline body weight did not differ among groups. Leucine supplementation with a high dose via drinking water (DEX-LH (H_2_O) group) significantly attenuated body weight loss when compared with the DEX-LL and DEX-LH (bolus) groups (*p* < 0.05). Soleus muscle mass was significantly reduced in DEX-LL (H_2_O) group when compared with the DEX-LL (bolus) and DEX-LH groups (*p* < 0.05). EDL muscle mass did not significantly differ among groups (Table 3).

**Table 3:** Body and muscle morphological parameters of the experimental groups.

Variable	Group			
	DEX-LL	DEX-LL (H_2_O)	DEX-LH	DEX-LH (H_2_O)
Initial BW (g)	442.3 ± 1.40	441.8 ± 1.58	444.9 ± 1.49	446.7 ± 1.27
Final BW (g)	338.9 ± 1.83	346.5 ± 3.58	340.0 ± 2.93	361.0 ± 4.13 ^a,b,c^
Delta BW (g)	−103.6 ± 2.07	−95.27 ± 3.14	−104.5 ± 3.35	−85.67 ± 6.77 ^a,b^
Soleus (mg)	222.5 ± 5.28	205.0 ± 2.39 ^a,b^	222.9 ± 5.54	207.4 ± 3.22
EDL (mg)	174.8 ± 2.79	170.5 ± 1.99	174.6 ± 4.12	169.4 ± 1.27

Values are expressed as mean ± SE. DEXA treated group plus low dose of leucine supplementation via gavage (DEX-LL; *n* = 10); DEXA treated group plus low dose of leucine supplementation via drinking water (DEX-LL (H_2_O); *n* = 10); DEXA treated group plus high dose of leucine supplementation via gavage (DEX-LH; *n* = 10). DEXA treated group plus high dose of leucine supplementation via drinking water (DEX-LH (H_2_O); *n* = 10). BW—body weight; EDL—extensor digitorum longus. ^a^ *p* < 0.05 *vs.* DEX-LL; ^b^ *p* < 0.05 *vs.* DEX-LH; ^c^ *p* < 0.05 *vs.* DEX-LL (H_2_O).

The effects of DEXA treatment and leucine supplementation on water intake: both DEX-LL (H_2_O) and DEX-LH (H_2_O) presented increased water intake at day 6 when compared with their intake at day 1 (48.80 ± 3.45 in DEX-LL (H_2_O) group at day 6 *vs.* 31.44 ± 3.09 at day 1; *p* < 0.05; 45.29 ± 1.84 in DEX-LH (H_2_O) group at day 6 *vs*. 34.25±2.93 at day 1; *p* < 0.05) and with the DEX-LL and DEX-LH at day 6 (34.67 ± 2.34 in DEX-LL group and 26.56 ± 3.98 in DEX-LH group at day 6; *p* < 0.05). The DEX-LH (H_2_O) group showed increased water intake when compared with the DEX-LL and DEX-LH groups at day 3 (33.08 ± 1.7 in DEX-LH (H_2_O) group *vs.* 23.14 ± 1.04 in DEX-LL group and 22.00 ± 1.98 in DEX-LH group; *p* < 0.05).

**Figure 4:**
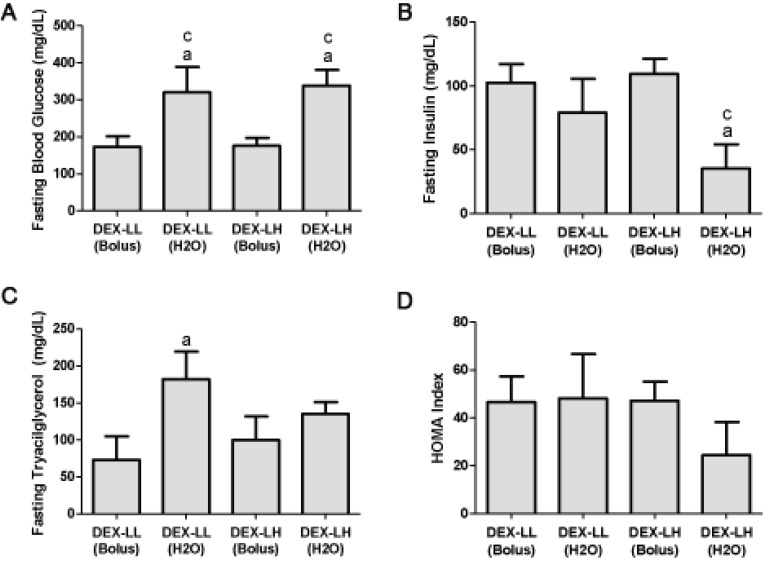
Fasting (**A**) blood glucose, (**B**) insulin and (**C**) triacylglycerol levels and (**D**) HOMA index. Values are expressed as mean ± S.E.M. ^a^ Different from DEX-LL (Bolus) (*p* < 0.05); ^b^ Different from DEX-LL (H_2_O) (*p* < 0.05); and ^c^ Different from DEX-LH (Bolus) (*p* < 0.05).

The effects of DEXA treatment and leucine supplementation on serum glucose, insulin and triacylglycerol (TAG): fasting serum glucose was significantly increased in the DEX-LL (H_2_O) group when compared with the DEX-LL group (320.3 ± 68.4 mg/dL in DEX-LL (H_2_O) group *vs.* 173.6 ± 28.2 mg/dL in DEX-LL group; *p* < 0.05; Figure 4A) and in the DEX-LH (H_2_O) when compared with the DEX-LH group (338.7 ± 41.9 mg/dL in DEX-LH (H_2_O) group *vs.* 176.1 ± 21.4 mg/dL in DEX-LH group; *p* < 0.05; Figure 4A). These results suggest a dose-response and administration route action of leucine. The DEX-LH (H_2_O) group also presented decreased fasting serum insulin when compared with the DEX-LH group (35.24 ± 18.94 mg/dL in DEX-LL (H_2_O) group *vs.* 109.3 ± 12.00 mg/dL in DEX-LL group; *p* < 0.05; Figure 4B). Regarding fasting triacylglycerol, DEX-LL (H_2_O) presented increased level when compared with the DEX-LL group (182.3 ± 37.1 mg/dL in DEX-LL (H_2_O) group *vs.* 72.2 ± 31.6 mg/dL in DEX-LL group; *p* < 0.05; Figure 4C). HOMA-IR index did not significantly differ among groups (Figure 4D).

At days 3 and 6 of the experiment (with the notable exception of the DEX-LH group at day 3), all groups presented markedly increased serum glucose level in the fed state when compared with their respective day 1 (*p* < 0.05). DEX-LL (H_2_O) and the DEX-LH (H_2_O) groups showed higher serum glucose level in the fed state when compared with the DEX-LH group at day 6 (*p* < 0.05; Table 4), suggesting that the route of administration promoted distinct results on blood glucose (*n* = 10).

**Table 4:** Fed serum glucose (mg/dL) of the experimental groups on days 1, 3 and 6 of the study.

Day	Group			
	DEX-LL	DEX-LL (H_2_O)	DEX-LH	DEX-LH (H_2_O)
1	104.8 ± 4.07	100.2 ± 4.14	114.7 ± 4.78	100.3 ± 4.95
3	196.5 ± 17.27 ^b^	175.4 ± 9.49 ^b^	178.1 ± 15.18	177.4 ± 10.16 ^b^
6	255.2 ± 23.44 ^b^	286.1 ± 37.34 ^a,b^	215.6 ± 22.33 ^b^	314.8 ± 39.34 ^a,b^

Values are expressed as mean ± SE. DEXA treated group plus low dose of leucine supplementation via gavage (DEX-LL; *n* = 10); DEXA treated group plus low dose of leucine supplementation via drinking water (DEX-LL (H_2_O); *n* = 10); DEXA treated group plus high dose of leucine supplementation via gavage (DEX-LH; *n* = 10). DEXA treated group plus high dose of leucine supplementation via drinking water (DEX-LH (H_2_O); *n* = 10). ^a^ *p* < 0.05 *vs.* DEX-LH in the same day of treatment; ^b^ *p* < 0.05 *vs.* day 1 in the same group.

**Figure 5:**
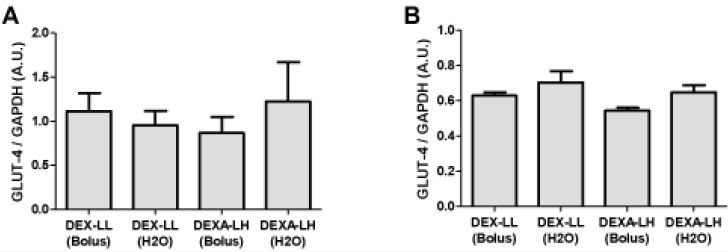
GLUT-4 gene expression in (**A**) soleus and (**B**) EDL muscles. Values are expressed as mean ± S.E.M.

The effects of DEXA treatment and leucine supplementation on GLUT-4 gene expression in skeletal muscle: no significant differences were recorded in GLUT-4 gene expression among groups (Figure 5; *p* > 0.05).

The effects of DEXA treatment and leucine supplementation on muscle functional parameters: no significant difference was observed in maximum ambulation, mean ambulation, maximum grip strength and mean grip strength among groups (*n* = 8–10).

## 4. Discussion

The major findings of the present study are that under DEXA treatment, leucine supplementation through gavage in both low and high doses was not capable of changing metabolic parameters (*i.e.*, triacylglycerol, fasting insulin levels and fasting glucose levels), but was capable of decreasing maximal voluntary strength function. On the other hand, when administered to leucine supplementated rats via drinking water and under DEXA treatment, even at low dosages, it was capable of inducing a massive diabetic state (and also decreasing the EDL mass), when compared with leucine supplemented rats via gavage, even at low doses. This result clearly demonstrates that not only the daily dosage, but also the administration form and leucine kinetics of this supplement are important players to be considered under DEXA treatment induced insulin resistance.

As previously described, leucine supplementation has been shown to spare skeletal muscle mass during several atrophic states, including insulin resistance [5]. Recently, it was demonstrated that supplementation with 0.6 g/kg of body mass of BCAA (46% leucine, 28% valine and 23% isovaline) was capable to attenuate the soleus muscle atrophy induced by DEXA (0.6 mg/kg given intraperitoneally—I.P., during 5 days) in Sprague Dawley rats [2]. However, in the same study, the authors did not report any information regarding insulin resistance. On the other hand, in our study, we chose the dosage of 5 mg DEXA/kg of body mass, since 1 mg/kg of body mass was unable to induce measurable skeletal muscle atrophy in our Wistar rats (data not shown). Moreover, when compared to previous studies of our group also using Wistar rats, DEXA given I.P. compared to DEXA given via drinking water [20], DEXA I.P. was slightly superior in increasing fasting glucose levels and inferior in inducing skeletal muscle atrophy [20]. From the above information, we conclude that: (1) glucose metabolism should be evaluated together with the possible sparing effect of leucine supplementation under glucocorticoid treatment; and (2) the administration pathway exerts a determinant effect on the magnitude of decrements in glucose homeostasis and skeletal muscle atrophy, not necessarily linked to each other.

As stated before, our major expectation was that leucine supplementation in low *versus* high dosages, due to its different physiological effects, would be capable of inducing profound changes in glucose homeostasis parameters, as well as skeletal muscle atrophy in both treated and non-treated DEXA groups. In fact, our expectation was that low dosages (due to the fact of non stimulating insulin secretion) [9] would be beneficial to DEXA treated rats, because glucose metabolism is already profoundly affected by glucocorticoid treatment. On the other hand, in energy restricted healthy rats, we were confident that high dosages would be better, because data from the literature suggests that higher leucine dosages would be more effective at decreasing muscle proteolysis [8]. Surprisingly, leucine supplementation offered via gavage was innocuous to glucose homeostasis and skeletal muscle mass under DEXA treatment in both low and high doses. This result demonstrated the capacity of the whole body to deal with low and even very high amounts of leucine administered as a *bolus*, which is somewhat surprising, because leucine, especially in high amounts, is capable of modifying insulin secretion (and consequently glucose uptake by peripheral tissues) and induces skeletal muscle protein synthesis, while decreasing muscle proteolysis [1,8,9,20]. Following the same line of reasoning and contrariwise to our initial hypothesis, in the healthy group (non DEXA treated group), both low and high doses showed similar effects on skeletal muscle atrophy. However, serum glucose, fasting insulin concentrations and also HOMA-IR index significantly decreased, suggesting an improvement in glucose metabolism in the low dose leucine supplemented group, which could be of clinical significance during weight loss diets, as suggested by Layman [21]. This result could explain why BCAA supplementation, in addition to dexamethasone treatment, was so effective in the treatment of skeletal muscle atrophy in the study conducted by Yamamoto and coworkers [2]. Interestingly, in a study of mice consuming high fat diet, the consumption of a chow containing 20% protein (with 1.5% leucine in w/v) increased oxygen consumption (and increasing resting energy expenditure) [5], and in C2C12 myocytes, leucine (0.5 mM) increased mitonchondrial mass by 30% and stimulated genes related to mitochondrial biogenesis [22]. Additionally, only leucine supplementation was able to protect animals from the deleterious effects of a high fat diet, such as insulin resistance and increased LDL cholesterol [5]. Although not directly measured in our study, we also observed a decrease in the blood TAG concentration in the low dose control group.

We then undertook a second study comparing the effects of DEXA plus leucine treatment with low and high doses of this amino acid used via bolus, as previously described, against the same daily concentration offered in the drinking water. Since the dosage effect was not different when comparing rats presenting insulin resistance mediated by DEXA treatment as shown in Figure 1A, would frequent nutritional stimuli be different from that provided by a pulsatile pattern to aggravate insulin resistance caused by DEXA treatment?

To test this hypothesis, we supplemented four groups of DEXA-treated rats, which consumed the same daily dose: the first two groups consumed the low dose of leucine in a pulsatile form (via gavage)*versus* a non-pulsatile form (via drinking water), and the second two groups followed the same schedule but consumed the high dosage. Our results were notable: rats supplemented through short periods of time (offered in drinking water), in a non-pulsatile form presented a markedly higher fasting glycemia compared with rats supplemented with the same daily dosage, in a pulsatile form (Figure 4A). These results suggest that tissues need time to terminate the leucine signal. Moreover, these results show that the continuous presence of this AA in the whole body, in an DEXA-induced insulin resistant state, would be capable of transforming to a clear diabetes state even with such a small leucine dose (*i.e.*, not capable of affecting glucose homeostasis when supplied via bolus). Importantly, this outcome also occurred in the high dosage group, which proves that the threshold of leucine supplementation capable of inducing diabetes, in a previous DEXA-induced insulin resistance, is extremely low when supplied via drinking water; this would be a completely novel result. On the contrary, the results clearly demonstrated that leucine supplementation with low dose via drinking water did not modify muscle mass of DEXA-treated animals when compared with gavage and the same pattern was observed with a high dose of leucine. This would mean that skeletal muscle at our end point, would not suffer the effects of this disturbance on glucose homeostasis. In fact, as shown below, GLUT-4 gene expression was unaltered in the muscles analyzed in this study. Interestingly, leucine supplementation (1.5% in drinking water for eight months) carried out in the polygenic mouse model NONcNZO10/LtJ (RCS10), which is predisposed to beta cell failure and type 2 diabetes, is able to improve the glycemic control that was associated with an increased insulin response to food challenge in control mice [23]. In our study, in the presence of DEXA plus high doses of leucine in the drinking water, we observed a significant decrease in the insulin level measured in fasted animals. Such a decrease may be associated with a failure of the beta cells to respond to high leucine concentration for insulin secretion in this group of animals. However, such an effect was not observed in the control animals supplemented with high leucine dose. Although insulin levels decreased in both situations, these results would indicate diametrically opposite situations. Under healthy conditions, a low dose of supplemented leucine would be capable of increasing glucose homeostasis and reducing insulin plasmatic levels. On the other hand, when given chronically at low and high doses in the presence of DEXA-induced insulin resistance, leucine supplementation promotes a clear diabetic state, and the diminishment of insulin levels observed with high doses would indicate a beta cell failure function. However, this hypothesis needs further research in order to be confirmed.

In muscle cells, glucose transport is mainly controlled by the stimulation of insulin, leading to the translocation of GLUT-4 from late and early endosome vesicles to the plasmatic membrane, as well as through control of gene expression. Indeed, multiple and complex mechanisms control the GLUT-4 transporter function [24]. In addition, it is acknowledged that DEXA treatment affects several steps of the insulin signaling cascade, leading to impaired glucose transport inside muscle cells [13]. However, there is very little information about the involvement of leucine supplementation in DEX-treated animals on GLUT-4 gene expression in different muscle tissues.

In study 1, we detected in EDL muscles a marked impairment of GLUT-4 gene expression in DEXA treated groups (Figure 2). EDL muscles are primarily composed of fast twitch muscle fibers [25]. This result may be linked with the modification of the genomic expression that can lead to impaired glucose transport [10,24]. This would mean that during conditions of supplementation with high doses of leucine and in short term periods, GLUT-4 expression is strongly controlled by hormonal inputs. In order to test such a concept, we compared in study 2 the effect of leucine supplementation in DEXA treated animals (Figure 5). The results obtained are compatible with the idea that with high doses of leucine in short-term periods, GLUT-4 expression is mainly controlled by hormonal inputs, not genetic ones. For example, Hu and coworkers [12] recently showed that under insulin resistant conditions (e.g., stressed rats showing increased glucocorticoid levels), the cortisol receptor binds to and inactivates the insulin receptor, demonstrating the strong impact of glucocorticoids during periods of insulin resistance on cell signaling. In another study by Doi and coworkers [26] examining the effects of isoleucine, the investigators found in C_2_C_12_ cells that the isoleucine effect on glucose uptake was mediated by phosphatidylinositol 3-kinase (PI3K). These results suggest that isoleucine stimulates the insulin-independent glucose uptake in skeletal muscle cells, which may contribute to the plasma glucose-lowering effect of isoleucine in normal rats. Collectively, our results suggest that healthy adult rats are capable of metabolizing very high amounts of leucine, and that the threshold of leucine supplementation needed to transform a protein synthesis signal into an insulin-resistant one is very high during normal states, but abnormal under glucocorticoid induced insulin resistance states, especially when supplied via drinking water.

The role of leucine supplementation in this scenario is uncertain because, when compared with Control-NS group, only Control-LL group showed a decreased GLUT-4 expression, and this decrease is evidenced only in the EDL muscle (Figure 2B). This result points out that GLUT-4 gene expression in EDL muscles may be altered not only by DEX treatment, but also by leucine supplementation, although this parameter is not predictive of changes in the whole body glucose metabolism and additional measurements, such as total GLUT-4, membrane-bound and glucose uptake in isolated muscles should provide more conclusive results.

Finally, when we evaluated the muscle function of these animals, we observed that animals treated with DEXA and receiving high dose of leucine presented a significant reduction in mean ambulation when compared with the control group. Surprisingly, control animals supplemented with high doses of leucine also presented lower mean grip strength when compared with the low dose group, suggesting that a high leucine dose, applied via bolus, is not innocuous in this experimental model after seven days of treatment.

## 5. Conclusions

These results show that the continuous presence of this AA in the whole body, in an insulin resistant state (DEXA-induced), would have several clinical implications: (1) will the results of prolonged leucine plus DEXA treatment in muscle cells (in a non-pulsatile form) apply to the whole body measurement? (2) Will patients receiving intravenous nutrition, but suffering from insulin-resistant states (induced by glucocorticoid treatment), benefit from AA supplementation? (3) Will skeletal muscle cells treated with glucocorticoids and essential AA have the same capacity to metabolize AA and glucose? (4) Are subjects that have resistance to insulin (due to overstress and hypercortisolemia induced stress) as showed by Hu and coworkers [12] and are ingesting several meals containing high amounts of protein (and leucine) per day capable of maintaining muscle mass and strength during these conditions? The answers to such questions are still unknown, and until further research clarifies the issue, the pros and cons will have to be weighed for each individual case.
